# Seven Months After Tropical Cyclone Chido in Mayotte: Early Lessons and Brain Health Challenges

**DOI:** 10.5334/aogh.4921

**Published:** 2025-11-06

**Authors:** Jacques Reis, Maxime Ransay-Colle, Alain Buguet, Farid Boumediene, Xavier Deparis, Peter S. Spencer

**Affiliations:** 1 Clinique neurologique, CHU Hôpital de Hautepierre, 2 Av Molière, 67000 Strasbourg, France; 2 Médecin de santé publique, Ex- conseiller médical du directeur général de l’ARS de Mayotte, France; 3 Malaria Research Unit, UMR 5246 CNRS, Claude-Bernard Lyon-1 University, 69622 Villeurbanne, France; 4 Faculté de Médecine de l’Université de Limoges, 2 rue du Dr Marcland, 87000 Limoges, France; 5 Agence régionale de santé de La Réunion, 97743 Saint-Denis cedex 9, France; 6 Department of Neurology, School of Medicine, Oregon Institute of Occupational Health Sciences, Oregon Health & Science University, Portland, OR 97239, USA

**Keywords:** extreme climatic events, risk perception, fatalities, violence, post-traumatic stress disorder, infantile PTSD

## Abstract

*Background:* On December 14, 2024, the tropical cyclone Chido hit the French department of Mayotte, an archipelago in the western Indian Ocean. It was the most violent storm in at least 90 years, causing widespread devastation.

*Objectives:* We address different aspects of the Chido disaster, including deaths, Mayotte’s vulnerabilities, and risk management, and review the hurricane’s health consequences, notably post-traumatic stress disorder (PTSD).

*Methods:* Information was collected through informal interviews with inhabitants, physicians, and stakeholders. Public data were provided by the public health authorities and through online searches.

*Findings:* Addressing pre-Chido vulnerabilities, risk perception, fatality assessment, community management, and post-Chido psychological consequence constitutes a major challenge for the Mayotte society.

*Conclusion:* We recommend launching an exploratory health study and planning to provide medico-psychological support to victims and to favor scientific investigations.

## Introduction

Chido, one of the around ten-yearly tropical cyclones [1] that move from east to west across the Indian Ocean, brought devastating winds, storm surges, and heavy rainfall that ravaged the two French islands (Maore and Pamanzi) of Mayotte on December 14, 2024 [2]. Considered the most severe regional cyclone in recent years, rating a four/five score on the Saffir–Simpson Hurricane Wind Scale [3, 4], Chido severely damaged infrastructure (e.g., buildings, airport, inter-island ferries, roads blocked by falling trees, etc.) [5], leading to major economic losses.

Health and notably brain health effects of extreme climatic events are rarely reported in Africa and the Indian Ocean zones. This article addresses the human health impact of Chido in Mayotte. We (JR) collected information through informal interviews with inhabitants, physicians, and stakeholders on the larger main island of Maore. Some official public data were provided by the public health authorities and by searching online.

### Cyclone chido and its death toll

On December 9, 2024, Chido started as a tropical storm southeast of the island of Diego Garcia in the Indian Ocean. Successively, the cyclone hit the Agaléga archipelago, Madagascar, and the Comoros archipelago (Comoros State), causing destruction but no casualties. On December 14, Chido devastated Mayotte, causing 40 deaths and 41 missing persons [6]. On December 15, Chido hit Mozambique, evolving to a tropical storm in Malawi and finishing its course in Zimbabwe [1]. The death toll was 120 in Mozambique and 13 in Malawi [7].

Following the World Meteorological Organization (WMO), cyclones are the “second-most dangerous natural hazards after earthquake” [8]. However, several articles underline the difficulty in tallying resulting death, whether in sub-Saharan cities [9] or even in the United States [10]. Difficulties in estimating casualties are obvious in displaced populations and migrants facing “complex humanitarian emergencies” [11, 12] and dealing with poverty and political issues [7]. In Mayotte, this difficulty is exacerbated by illegal immigration.

### Singularity and vulnerability of mayotte

Mayotte, France’s 101st department since March 2011, faces numerous challenges: cultural, socio-economic, and political [13, 14]. In addition to an infrastructure ill-adapted to this cyclone-prone area, major public health concerns, such as poverty, insecurity, an insufficient healthcare structure, and poor health conditions, contribute to Mayotte’s specific vulnerability. For example, some demographic issues challenge Mayotte’s Mahoran society, where population growth is the highest in France. Between 2014 and 2024, the Mahoran population increased by 43%. Foreigners represented 48% of the population in 2017. In 2024, 53.8% of the habitants were <20 years of age [15]. Since 2010, inhabitants have suffered from many socio-economic crises, a water-access crisis, and disease outbreaks, including waterborne (e.g., typhoid, cholera), respiratory (whooping cough), and arthropod-borne viral disorders (notably dengue) [16].

### Risk management and perception

Chido’s trajectory was correctly monitored [17] and the risks carefully evaluated by the “Centre météorologique régional spécialisé” (CMRS) based on Réunion Island, and operated by Météo-France. A cyclone pre-alert was issued on Wednesday, December 11, at 3 p.m. local time. Recommendations were duly issued via the media and cellphone messaging, and on Friday the 13th, imams warned their faithful in Mayotte’s 277 mosques. However, a portion of the population, notably slum dwellers, did not conform to the authorities’ recommendations regarding the cyclonic risk. The probability of Chido hitting Mayotte was considered very low [4], and popular memory only recalled the major and disastrous 1934 cyclone [17]. In addition, previous recent cyclones had spared Mayotte and were far less intense than in neighboring territories; only seniors remembered the cyclones of 1984 and 1985.

Even in countries prone to extreme events, population preparedness for severe weather events presents a significant challenge. Preparedness, mental and psychological awareness are important factors for coping with traumatic events [18]. An interview-based American study described the general public’s poor compliance with evacuation orders for Hurricanes Katrina and Rita that hit the United States Gulf Coast in August and September 2005 [19]. Two reasons emerged from victim interviews: their lack of “trust in the reliability of the forecast” and “the belief their home can safely survive the storm.” A second victim group pointed to a “need of specific information” and a lack of “services to aid them in their evacuation efforts” [19]. Management of the 2013 Typhoon Haiyan in the Philippines revealed concerns regarding early-warning systems to communicate threats of rare, extreme events [20]. Underestimation or ignorance can result in low adhesion to recommendations and an absence of safety-seeking behavior. This was the case in Mayotte, contrary to the Philippines and the Caribbean Island of Dominica that are “accustomed to regular hazards causing mild to moderate damages” [21].

### Management of the disaster and post-chido health disorders

The Chido disaster response was planned locally and at the national level. Rescue teams (notably the Gendarmerie) quickly restored road traffic, allowing access to victims, provision of first aid, and dispatch of food and bottled drinking water. Some days later, when air transportation was again possible, Metropolitan France and Réunion Island supplied further aid. One major concern was lodging persons who had lost their dwellings; mosques and schools provided shelter for the disaster-stricken population. Another challenge was the insecurity and violence (vandalism and depredation, plundering) [22] in the immediate post-Chido period. Domestic violence also increased, particularly in precarious families.

The island’s hospital in Mamoudzou (Mayotte prefecture) was damaged but the remarkable local health professionals continued providing care; a total of 9,798 outpatients visited the hospital’s emergency ward between December 14, 2024 and February 16, 2025 [23]. Additionally, France’s Civil Security (*Élément de Sécurité Civile Rapide d’Intervention Médicale*) deployed an Emergency Medical Team to Mayotte that set up a field hospital that provided care for 6,303 outpatients and 146 inpatients between December 24 and February 3, 2025 [23]. During the first post-Chido week, the first reason for care was obviously physical trauma (fractures, wounds, contusions, foreign bodies), followed one week later by superinfected wounds. Some cases of waterborne disease (acute viral gastroenteritis, typhoid, cholera) were seen in the following weeks [23].

In addition to local resources, psychological support teams were active during the post-Chido phase. Health professionals of the “Cellule d’Urgence Médico-Psychologique” were deployed to Mayotte between December 18, 2024 and February 7, 2025. Psychologists from the NGO “Terra Psy - Psychologues Sans Frontières” set up counseling in schools and remain in action. A March 2025 epidemiological study employed the DSM-5 (PTSD checklist for DSM-5, PCL-5) 20-item self-report questionnaire [24] and collected 302 responses; results showed that post-traumatic stress disorder (PTSD) prevalence was 36% for nationals, 51% for legal foreigners, and 77% for illegal migrants. The highest PCL-5 score was 69%, among men [25].

While anecdotal, some observations are of great interest and therefore should be investigated. One of JR’s colleagues, a general practitioner (RL), observed a post-Chido break in the growth curve of a monitored infant, this sign being confirmed in other children by another professional (JD). A college supervisor reported a post-Chido change in the behavior of young adolescents who had become “quiet,” and noted the departure from Mayotte of high-school students (quantitative data to be searched) for La Réunion or Metropolitan France. The psychological impact on babies, children and young adolescents is a major concern for mental health.

### Brain health consequences of extreme events

As pointed out by Schmidt, “cyclone exposure [can lead] to mental health problems, especially post-traumatic stress disorder,” with an elevated PTSD risk even 2 to 3 years after the event [26].

The psychological effects (depression and PTSD) of extreme events, notably cyclones, have been known for three decades [27, 28], although among all health effects (e.g., drowning, injuries), they were not discussed in an historical review of tropical events that occurred between 1980 and 2009 [21]. Interest in mental effects related to natural disasters, in particular cyclones, is shown by recent systematic reviews and meta-analyses [29, 30]. PTSD is the prevalent condition associated with a wide range of traumatic events [31]. Being exposed to and/or a victim of a cyclonic event represents a major risk factor for PTSD. The risk is augmented for pregnant women, high-school students, and public health workers [29, 32]. Pregnant women exposed to a cyclone have an increased risk for preterm birth, and the babies have lower birth weights [29], which has been associated with increased risk for certain physical (diabetes, hypertension, metabolic syndrome) and mental health issues later in life. PTSD risk also seems to be gender-related as “women appear to be more likely to suffer psychological distress (e.g., anxiety, PTSD, gender-based violence) than men” [29, 32]. An elevated risk for PTSD following a cyclone exposure was observed with a delay of six months to two years [29]. The occurrence and notably the severity of PTSD also depend on a preexisting personal vulnerability [33] and sociologic conditions, e.g., displacement [34]. A very important concern is that investigations on mental health and psychological trauma after extreme weather events are mostly restricted to affected populations of high-income countries. Specifically, Africa lacks research in this field [26, 29, 35].

PTSD occurring in children and adolescents has been extensively scrutinized in high-income countries [36–45]. Although a consensus is lacking regarding all criteria for the clinical diagnosis [40], specific tests for pediatric PTSD have been developed for school-age children (over 6–7 years) and adolescents [40, 41], especially since children do not spontaneously report their trauma experiences [41]. While for children the psychological impact with respect to the age of trauma exposure is unclear, in adults, “trauma exposure has been shown to about double the risk for major depressive disorder” [42]. A stressful experience occurring before the age of five years increases the risk of anxiety in adulthood [43].

There is an important lack of knowledge regarding the effects of trauma in early childhood, infants (<1 year old), and preschool-aged children (1–5 years old) [42, 45]. An historical article addressed the health effect of Hurricane Gilbert that struck Jamaica in 1988, where deficient growth in a 23–44-month-old infant cohort was attributed to food shortage [45]. Early age is obviously a critical window if one considers the Developmental Origins of Health and Disease hypothesis [46] and experimental data concerning epigenetic modifications related to perinatal stress in animals [47].

The psychological consequences of any disaster, especially related to extreme weather events, are obviously increased by additional stress induced, for example, by violence and out-of-home insecurity. The stress effect of violence is well documented, particularly for women and girls [48], and also between intimate partners [49].

Psychological distress and PTSD also have a major impact on brain health, of which the effect on sleep quality is well known. In the City of Tacloban (Philippines), a study of two groups of survivors, 18 and 30 months after Typhoon Haiyan, demonstrated an association of PTSD with insomnia, nightmares, and poor sleep quality [50]. A polysomnographic and actigraphic evaluation of 3–8-year-old children with PTSD revealed “higher sleep fragmentation, increased wake-after-sleep onset, increased number of sleep stage changes,” and sleep-rhythm disturbances compared to controls [51]. The bidirectional relation between sleep and PTSD is an important issue [52] explaining why sleep quality is a major determinant of brain health [53]. Among the long-term effects, the consequences of stress concern the autonomic nervous system in adults [54] and youth [55] and the immune system [56]. Another mental effect must be considered in adolescents, namely the potential for radicalization that endangers the societal structure [57].

## Conclusions

Several important concerns are underlined here: pre-Chido vulnerabilities, risk perception, and post-Chido community behavior. Chido is an example of a population-wide traumatic experience that may impede long-term societal health and vulnerability. The cyclone had a massive impact on Mayotte by creating a stupor that cannot be explained by fatalism and treated solely by considering the inhabitants’ resilience. The disaster also probably reawakened ancient cyclone-related traumatic experiences and increased violence in Mayotte’s complex and composite society [58].

Post-Chido psychological consequences constitute a major challenge for the Mayotte society. While effective treatment and long-term follow-up of adult patients with PTSD is mandatory, our main concern is treating and investigating infants and children to assess long-term psychological and physical consequences on brain health [59] in collaboration with their parents and relatives.

Because of the ongoing climate change, cyclones are expected to recur earlier in the season, become more intense [60], and impact areas that previously have been spared or moderately affected. Experience, feedback, and lessons from the Chido cyclone could serve to improve preparedness, disaster management, primary disease prevention, and financial assistance planning [44] in populations facing cyclones, especially in Indian Ocean regions prone to such extreme natural events. We recommend launching an exploratory health study with the aim of providing medico-psychological support for victims and favoring scientific investigations.
